# Abuse and Use of Atropia in Eye Treatment

**Published:** 1895-09

**Authors:** Richard H. Satterlee

**Affiliations:** Buffalo, N. Y.


### ABUSE AND USE OF ATROPIA IN EYE TREATMENT.

By RICHARD H. SATTERLEE, M. D., Buffalo, N. Y.

I HAVE recently had two cases which are instructive as being so typical of what often comes to the oculist that I would like to call the attention of the profession to them.

CASE I. Mr. B. gives the following history: he noticed his sight failing gradually for about six months, then he consulted his family physician. The physician examined his eyes superficially and used a solution of atropia. The patient was blind instantly. The sight partially returned in two days, when atropia was used again, and this time the sight did not return. I saw the case later and found glaucoma present in both eyes. I operated, but there was only slight improvement of vision.

CASE II. I have under my care, at present, a woman about thirty years of age, who had an attack of rheumatic iritis two years ago. She was under the care of her family physician during this attack, whose whole treatment consisted of confinement in a dark room for three months, and weak solutions of nitrate of silver and sulphate of zinc dropped in the eyes three or four times daily. On examination, I found the iris of both eyes bound down so firmly by posterior synechia that no mydriatic had any appreciable effect. She has asthenopia that glasses will only partially relieve. The synechia has interfered with the nutrition of the lens, and it is only a question of a short time when she will have cataracts in both eyes.

In case I. the sulphate of atropia was positively contraindicated, for the angle of filtration was already partially closed, and dilatation of the iris completed the closure, greatly increasing the pressure inside the eye, and thus doing still further injury to the optic nerve. There was no redness of the conjunctiva, nor discoloring of the iris, indicating a need of any mydriatic. The indiscriminate use of atropia is dangerous, for it must be remembered that glaucoma may occur at almost any age after puberty and, following injury, even younger, I myself having had a case, four years ago, of glaucoma in a boy nine years old.

In case II. the symptoms all pointed to the need of atropia of sufficient strength to produce dilatation of the iris and so avoid, if possible, the formation of synechia. Synechia will sometimes form in spite of all treatment, but atropia rather tends to hinder the formation, by bringing the iris to a state of rest and thus lessening the inflammation.

189 Delaware Avenue.

